# Correction: Biochemical and Cellular Evidence Demonstrating AKT-1 as a Binding Partner for Resveratrol Targeting Protein NQO2

**DOI:** 10.1371/journal.pone.0106137

**Published:** 2014-08-18

**Authors:** The PLOS ONE Staff

## Notice of Republication

This article was republished on July 25th, 2014, to include missing panels from several figures. The publisher apologizes for this error. Please download this article again to view the corrected version. The originally published, uncorrected article and the republished, corrected article are provided here for reference.

## Supporting Information

File S1 Originally published, uncorrected article
(PDF)

File S2 Republished, corrected article
(PDF)
