# Supplementary material for: Correction: Biochemical and Cellular Evidence Demonstrating AKT-1 as a Binding Partner for Resveratrol Targeting Protein NQO2
Source: PLoS One. 2014 Aug 18;9(8):e106137. doi: 10.1371/journal.pone.0106137 (PMC4136916; doi:10.1371/journal.pone.0106137)
Supplement: File S2 — Republished, corrected article (PDF) [file pone.0106137.s002.pdf]

Biochemical and Cellular Evidence Demonstrating AKT-1 as a Binding Partner for Resveratrol Targeting Protein NQO2

Tze-chen Hsieh[1]*, Chia-Yi Lin[2], Dylan John Bennett[1], Erxi Wu[3], Joseph M. Wu[1]

1 Department of Biochemistry and Molecular Biology, New York Medical College, Valhalla, New York, United States of America, 2 Genome and Systems Biology Degree Program, National Taiwan University, Taipei, Taiwan, Republic of China, 3 Department of Pharmaceutical Sciences, North Dakota State University, Fargo, North Dakota, United States of America

Abstract

Background: AKT plays an important role in the control of cell proliferation and survival. Aberrant activation of AKT frequently occurs in human cancers making it an attractive drug targets and leading to the synthesis of numerous AKT inhibitors as therapeutic candidates. Less is known regarding proteins that control AKT. We recently reported that quinone reductase 2 (NQO2) inhibited AKT activity, by unknown mechanisms.

Methodology/Principal Findings: In this study, molecular modeling was used to query interaction between NQO2 and AKT. We found that pleckstrin homology (PH) and kinase domains of AKT bind to chains A and B of NQO2. Pull-down and deletion assays revealed that PH domain of AKT is essential for interaction with NQO2. Modeling analysis further revealed that kinase domain of AKT binds NQO2 in the vicinity of asparagine 161 located in the resveratrol-binding domain of NQO2. In studies to test whether exposure to resveratrol potentiates or diminishes AKT binding to NQO2, we showed that pre-binding by resveratrol in wild type but not histidine-161 (N161H) mutant NQO2 significantly affected this interaction. To obtain information on interplay between resveratrol and AKT, resveratrol affinity chromatography was performed. AKT binds with high affinity to the column suggesting that it is a target of resveratrol. The half-life of AKT mRNA decreased from ~4 h in control cells to ~1 h in NQO2-knockdown cells. The inhibition of AKT by resveratrol was attenuated in NQO2-expressing relative to NQO2-knockdown cells.

Conclusion/Significance: Both NQO2 and AKT are targets of resveratrol; NQO2:AKT interaction is a novel physiological regulator of AKT activation/function.

Citation: Hsieh T-c, Lin C-Y, Bennett DJ, Wu E, Wu JM (2014) Biochemical and Cellular Evidence Demonstrating AKT-1 as a Binding Partner for Resveratrol Targeting Protein NQO2. PLoS ONE 9(6): e101070. doi:10.1371/journal.pone.0101070

Editor: James Porter, University of North Dakota, United States of America

Received February 10, 2014; **Accepted** June 2, 2014; **Published** June 26, 2014

Copyright: © 2014 Hsieh et al. This is an open-access article distributed under the terms of the Creative Commons Attribution License, which permits unrestricted use, distribution, and reproduction in any medium, provided the original author and source are credited.

Funding: This research was supported by the Intramural Sponsored Research Program of New York Medical College to T.C.H. (49-432-1 and 49-466-1). The funders had no role in study design, data collection and analysis, decision to publish, or preparation of the manuscript.

Competing Interests: The authors have declared that no competing interests exist.

* Email: Tze-chen_Hsieh@nymc.edu

Introduction

AKT, a serine-threonine kinase that is involved in a variety of cellular processes including cell survival, proliferation, metabolism, and response to inflammatory agents [1–4], is aberrantly activated in correlation with oncogenic transformation and tumor growth. Elevated AKT occurs in ~50% of all human cancers including prostate cancer (CaP) [5,6] and its activation is subject to negative regulation by tumor suppressor phosphatase and tensin homolog (PTEN) [5,7]. Loss of PTEN occurs at high frequency in high-grade and metastatic CaP and is accompanied by constitutive activation of AKT [8], attesting to the critical role AKT plays in prostate carcinogenesis [9,10]. Yet, up to 30% of recurrent castration-resistant tumors are also PTEN-positive [11,12]. The incongruent findings suggest that endogenous proteins might exist capable of regulating and modulating the expression, activation and function of AKT.

Epidemiologic studies demonstrate an association between consumption of diet rich in fruits and vegetables with reduced risk of developing many cancer types; furthermore, dietary grape polyphenol resveratrol has been shown to inhibit AKT activity [13–17]. However, it is not known how resveratrol controls AKT, particularly in the context of its reported anti-CaP activity. NQO2 is an oxidoreductive enzyme that utilizes dihydronicotinamide riboside (NRH) as a co-substrate for converting quinones to hydroquinones, and is traditionally considered as a phase II detoxification enzyme. NQO2 has been identified by us as a resveratrol target protein [18] and its participation in chemoprevention by resveratrol is supported by our recent studies showing that resveratrol mediates NQO2-dependent cyclin D1 degradation in CWR22Rv1 CaP cells [19]. AKT phosphorylates glycogen synthase kinase 3 (GSK-3α/β) at serine 21/9 (Ser21/9). This phosphorylation deactivates GSK-3 leading to a decrease in cyclin D1 phosphorylation at threonine 286 (Thr286) and subsequently cyclin D1 accumulation [20,21]. However, details by which NQO2 acts on AKT activation/deactivation remain largely unknown.

Previously it has been reported that physiologically achievable concentrations of BCR-ABL kinase inhibitors bind and inhibit both NQO2 and ABL activities [22,23]. Since we found that NQO2-knockdown cells showed an accompanying increase in AKT activity [19], we consider that NQO2 could decrease AKT activity through its direct binding to AKT and in addition, possibly by forming a complex with resveratrol. To test this hypothesis, in the present study, we employ biochemical and cellular assays in combination with *in silico* modeling to examine a hitherto unreported interaction between NQO2 and AKT and to unravel the modulation of this interaction by resveratrol. We showed that (i) AKT is a binding partner for NQO2; (ii) interaction between NQO2 and AKT is directed at the PH domain of AKT; (iii) resveratrol affects the interaction between NQO2 and AKT and (iv) AKT is a newly discovered resveratrol target protein. Our results reveal a novel control of AKT by non-kinase NQO2 and that NQO2 participates in resveratrol-induced anti-CaP activity by targeting AKT/GSK-3β/cyclin D1 mediated growth control.

Materials and Methods

Reagents

Epoxy-activated agarose resin (12 atom linker, 33 μmol of epoxy group/ml of packed gel) and resveratrol were purchased from Sigma-Aldrich Corp. (St. Louis, MO, USA). Stock of resveratrol (12.5 mM) was prepared in dimethyl sulfoxide (DMSO) and kept at −20°C. The human NQO2 and N161H NQO2 recombinant proteins were provided by Dr. Z. Zhang and its preparation was detailed previously [18]. The human AKT recombinant protein was obtained from Upstate Biotechnology, Inc. (Lake Placid, NY, USA). All other chemicals and solvents used were of analytical grade and purchased from various commercial vendors.

Cell culture

Human K562, PC-3 and CWR22Rv1 cells were obtained from American Type Culture Collection (ATCC, Rockville, MD, USA). CWR22Rv1 cells containing stably expressed shRNA-mediated NQO2-knockdown were established using procedures described [19]. Single colonies were picked for propagation and expansion. Using this approach, a number of stable monoclonal cell lines were established. The NQO2 knockdown was confirmed by immunoblot analysis. The two sublines used for this study were designated shRNA08 and shRNA25, representing the control and NQO2-knockdown CWR22Rv1 cells (∼53% decrease in NQO2 expression), respectively [19]. Routinely, cells were cultured in RPMI-1640 media containing L-glutamine, supplemented with 10% FBS, penicillin (100 U/ml), and streptomycin (100 μg/ml) (Cellgro, Inc., Herndon, VA, USA). Cells were split once a week and media were changed every 3–4 days.

Preparation of cell extracts and Western blot analysis

Cells were collected and lysed on ice for 30 min in cold immunoprecipitation (RIPA) buffer, which contained 50 mM Tris, pH 7.4, 150 mM NaCl, 1 mM EDTA, 1% Triton X-100, 1% deoxycholate, 0.1% SDS, 1 mM dithiothreitol and 10 μl/ml protease inhibitor cocktail from Sigma-Aldrich Corp. (St. Louis, MO, USA). Protein concentrations were determined by Coomassie protein assay kit (Pierce, Rockford, IL, USA). Proteins were separated by 10% SDS-PAGE followed by Western blot analysis using specific primary antibodies. The expression of actin was used as a loading control. Immunoreactive bands were detected by enhanced chemiluminescence (ECL) (Kirkegaard & Perry Laboratories, Inc., Gaithersburg, MD, USA). The intensity of the specific immunoreactive bands was quantified by densitometry and expressed as a ratio relative to the expression of actin [24,25]. The primary antibodies for AKT and NQO2 were from Cell Signal Technology, Inc. (Beverly, MA, USA) and Santa Cruz Biotechnology, Inc. (Santa Cruz, CA, USA), respectively.

Gel filtration chromatography

CWR22Rv1 cell lysates containing 400 mg of protein were loaded on a Superose 12 HR 10/30 column (Amersham Biosciences Corp., Piscataway, NJ, USA) and run on a FPLC system. The column was equilibrated with buffer (10 mM Tris pH 7.5, 150 mM NaCl) and the elution was carried out at a flow rate of 0.25 ml/min; fractions of 0.25 ml were collected and aliquots were analyzed by SDS-PAGE and Western blotting as described above.

Co-localization study and analysis by fluorescence microscopy

PC-3 cells were cultured in RPMI-1640 with 10% fetal bovine serum (FBS) for 72 h. Cells were fixed with 3.7% formaldehyde. Fixed cells were air dried, and then washed with PBS followed by incubation with PBS containing 10% BSA. Next, cells were incubated with anti-NQO2 goat IgG (Santa Cruz Biotechnology, Inc., Santa Cruz, CA, USA), anti-AKT rabbit IgG (Cell Signaling Technology, Inc., Beverly, MA, USA) followed by incubation with FITC- or rhodamine-conjugated secondary antibodies and DAPI (40,6-diamidino-2-phenylindole) (Sigma-Aldrich Corp., St. Louis, MO, USA), and then examined using fluorescence microscopy. A Zeiss microscope equipped with Axiovert 200 Imaging System (Carl Zeiss MicroImaging Inc., Jena, Germany) was used to capture cell images at 200X magnification.

Immunoprecipitation (IP)

The human NQO2 or AKT recombinant proteins were incubated in IP-buffer (50 mM Tris-HCl, pH 8.0, 100 mM KCl, 0.1% NP-40, 2 mM EDTA, 1 mM phenylmethylsulfonyl fluoride (PMSF) and protease inhibitor cocktail) with NQO2 antibody overnight at 4°C. The protein A/G-Sepharose beads (Santa Cruz Biotechnology, Inc., Santa Cruz, CA, USA) were added and incubated for 2 h at 4°C, followed by washing five times with buffer containing 50 mM Tris-HCl, pH 8.0, 150 mM NaCl, 0.5% NP-40, 2 mM EDTA and 1 mM PMSF. The proteins bound to the beads were analyzed by separation on 10% SDS-PAGE followed by Western blot analysis as described above.

Fusion protein purification and pull-down assays

GST fusion or His-tagged, NQO2 or AKT proteins were expressed in *Escherichia coli* BL21DE3 (*plys*) and purified by the use of either glutathione beads (Amersham Biosciences Corp., Piscataway, NJ, USA) or nickel-nitrilotriacetic acid agarose (Qiagen Inc., Valencia, CA, USA). GST-NQO2 fusion proteins (up to 2.5 μg) were incubated with His-tagged AKT proteins (up to 2.5 μg) in GST binding buffer (50 mM Tris-HCl, pH 7.8, 1 mM EDTA, 150 mM NaCl, 1% Nonidet P-40 and 0.2 mM PMSF). The reaction mixtures were incubated by gentle rocking for 2 h at 4°C, immobilized on glutathione-Sepharose beads for another 2 h at 4°C, and washed five times with binding buffer. The bound proteins were separated on 10% SDS-PAGE followed by Western blot analysis using anti-His (Sigma-Aldrich Corp., St. Louis, MO, USA), anti-AKT or anti-NQO2 antibodies as probes.

Preparation of resveratrol affinity column and fractionation of cytoplasmic extracts on resveratrol affinity columns

Resveratrol was immobilized on epoxy-activated agarose as described [26]. In brief, one gram of epoxy-activated agarose was suspended in ice-cold water for 5 min and washed extensively to remove the preservatives. Resveratrol (23 mg) dissolved in 2.5 ml of 0.1 M NaOH was added to 1 ml of resuspended epoxy-activated agarose, followed by an overnight incubation at room temperature to allow chemical coupling of resveratrol to the resin. To stop the reaction, 6 ml of 1 M sodium acetate buffer (pH 5.0) containing 1 mM dithiothreitol (DTT) was added to the mixture to neutralize unreacted epoxy groups and prevent further oxidation of resveratrol. Immobilized resveratrol resin was washed successively with 0.1 M sodium acetate, pH 5.0, containing 1 mM DTT and 70%, 30%, 10%, and 0% ethanol, respectively. Cultured K562 cells were lysed and 200 µl cell extract containing 600 µg protein was passed through resveratrol affinity column to analyze the resveratrol target/binding protein profiles as described previously [26,27]. The column was eluted 5–7 times, each time with 0.5 ml lysis buffer containing 0.35 M NaCl. This was followed by the same number of washing using 1 M NaCl supplemented buffer. Next, the column was equilibrated with the lysis buffer and eluted with 0.5 ml 1 mM ATP. After the column was extensively washed with 1 mM ATP supplemented buffer, the column was again re-equilibrated with the lysis buffer and eluted with 1–2 mM resveratrol dissolved in 2% DMSO as the last step. Eluted proteins were concentrated by precipitation with methanol-chloroform-water [28], separated on 10% SDS-PAGE, visualized by silver staining and analyzed by Western blot analysis [27,29].

Determination of AKT mRNA expression by RT-PCR

Total RNA was extracted using the TriZol reagent (Invitrogen, Carlsbad, USA). Isolated RNA (0.5 µg) was amplified by PCR using one-step RT (reverse transcriptase)-polymerase chain reaction kit (Promega Corp., Madison, WI, USA). The PCR primer sequences used were: AKT, forward 5′- CAC ACC ACC TGA CCA AGA TG -3′, reverse 5′- CCT CAG AGA CAC GGC CTT AG -3′; GAPDH, forward 5′-CCA CCC ATG GCA AAT TCC ATG GCA -3′, reverse 5′-TCT AGA CGG CAG GTC AGG TCC ACC -3′. PCR condition for AKT mRNA expression: denaturation, 95°C, 5 min, followed by 30 cycles of denaturation at 94°C for 1 min, annealing at 60°C for 1 min, and extension at 72°C for 1 min. Correspondingly, PCR condition for GAPDH: denaturation at 95°C, 5 min, followed by 28 cycles of denaturation at 95°C for 30 s, annealing at 60°C for 30 s, and extension at 72°C for 30 s. The expression of GAPDH was used as a control for normalizing AKT mRNA levels.

Protein modeling

Known 3D protein structure files were obtained from the RSCB Protein Data Bank (PDB) in the PDB file format for NQO2 (PDB code: 1SG0) and AKT (PDB code: 3O96). The files were then prepared in UCSF Chimera [30] by removing ligands and uploaded to GRAMM-X Protein-Protein docking server [31,32] for analysis. The top energy scoring output containing both proteins was analyzed using UCSF Chimera to identify clashes and hydrogen bonds formed between two proteins as well as to visualize the crystal and surface structure of the bound proteins [33].

Docking Analysis

UCSF DOCK6.3 programs were used to perform Docking as described [34]. All ligands used for the docking experiments were obtained from the UCSF ZINC database in mol2 format. ZINC provides the ligands in fully charged and docking ready format for uploading into UCSF docking server. The known X-ray structure of AKT in complex with inhibitor VIII (PDB code: 3O96) was used as a reference receptor structure. The total binding energies were calculated. Ligands with low binding energies were identified as likely to have a high affinity towards the receptor. Once docking was completed, results were analyzed using the UCSF program Chimera and viewed by Chimera's ViewDock tool [30]. Chimera's Find H-Bond tool was used to determine hydrogen-bonds the ligand may form with the receptor. The angstrom cutoff for H-bonding was relaxed by 0.4 angstroms that allowed bonding up to 3.9 angstroms. All docking procedures were performed in a shell program using a Linux based machine.

Procedure for docking of small molecule ligands to multiple protein:protein complexes

To test whether and how the presence of a ligand, such as resveratrol, bound to a receptor (target protein) could affect protein-protein binding characteristics, the ligands obtained from the ZINC database were first docked with UCSF DOCK6 prior to the protein-protein binding simulation. Once these calculations were completed, structures with the ligand present were prepared, consolidated using UCSF Chimera's "DockPrep" tool and saved as PDB files. For NQO2, dockings with DOCK6 was performed twice, once for active site of each monomer, in order to place the ligand in active sites of both NQO2 monomers. Once the structures were prepared, they were uploaded to PatchDock for match-principle docking [35]. The results from PatchDock were then sent to FireDock for refinement and scoring [36,37] which yielded the protein-protein interaction (PPI) score readout. The best scoring model from each of the complexes were then retrieved and downloaded from FireDock and re-prepared for docking in Chimera. Ligands were then allowed to dock with these structures to determine how protein binding could affect interaction with small molecules. In the analysis of certain models where the presence of another bound protein on small molecule binding was not the focus of the analysis, a ligand was simply docked by the aforementioned process, except that the preparation was uploaded as a single structure in Chimera followed by docking and analysis. PatchDock/FireDock was utilized for our analysis as they contained both scoring features and the capacity for docking with ligands.

Results

Evidence that interaction occurs between NQO2 and AKT

Previously, we reported an increase in AKT activity in NQO2 knockdown CWR22Rv1 cells [19], raising the possibility that NQO2 might form a complex with AKT and thus providing a mechanism that may sequester AKT and prevent its functional activation. To test this hypothesis, GRAMM-X molecular modeling was used to analyze the potential that NQO2 interacts with AKT. Structure code for NQO2 (1SG0) and AKT (3O96) were obtained as PDB files and uploaded for analysis. The results showed that four hydrogen bonds can form between these two proteins: Asp133 in chain A of NQO2 forms two hydrogen bonds with Arg15 and Lys20 located in PH domain of AKT, while Thr156 and Thr158 in chain B of NQO2 likely hydrogen bond with Glu322 and Asn324 found in kinase domain (KD) of AKT (Fig. 1A). Prediction of a complex between NQO2 and AKT

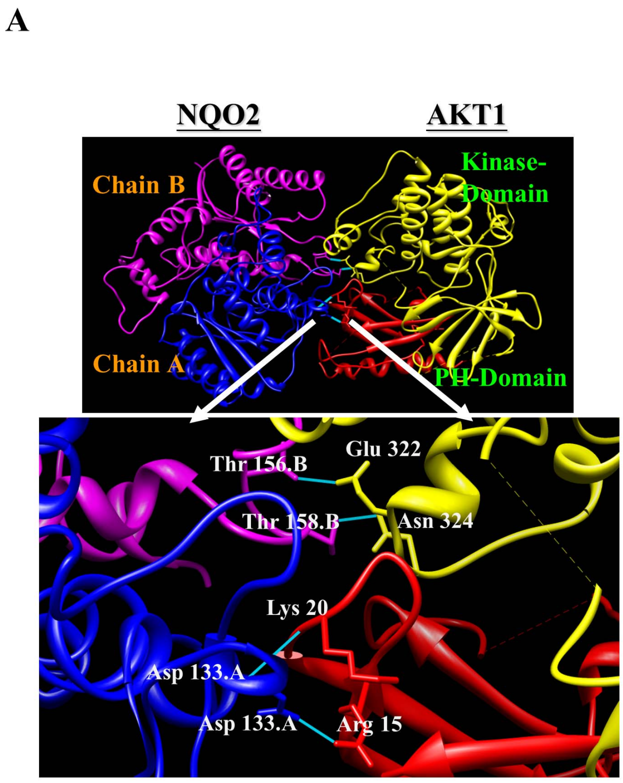

NQO2 Amino Acid Residue (.Chain)	AKT1 Amino Acid Residue (domain)	Hydrogen Bond Length
Asp 133.A	Arg 15(PH)	3.336 A
Asp 133.A	Lys 20(PH)	2.917 A
Thr 158.B	Asn 324(KD)	2.922 A
Thr 156.B	Glu 322(KD)	3.296 A

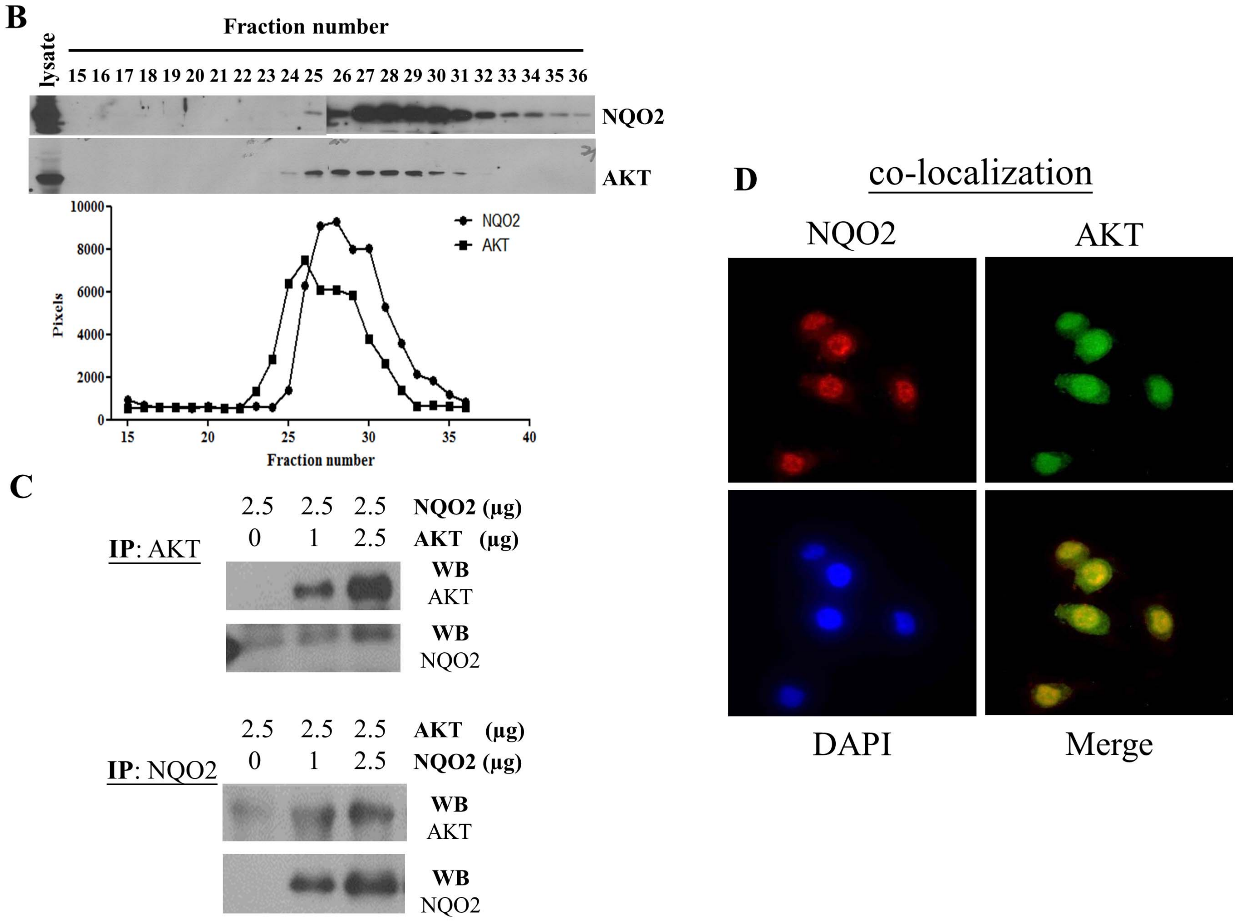

Figure 1. Protein-protein interaction between NQO2 and AKT. (**A**) GRAMM-X molecular modeling analysis was used to examine the possible interaction of NQO2 (PDB: 1SG0) and AKT (PDB: 3O96). The output data from the GRAMM-X program showed the potential interaction between these two proteins. H-bonds formed between proteins are shown as blue solid lines and the distance of H-bonds between amino acid residues are presented as angstrom (Å). (**B**) Gel filtration (technical advice provided by Dr. Z. Zhang) was performed to examine complex formation between NQO2 and AKT. CWR22Rv1 cell lysates were applied to a Superdex 200 column and eluted fractions were analyzed by western blotting. (**C**) Wild-type NQO2 and AKT recombinant proteins were immunoprecipitated with anti-NQO2 or anti-AKT antibodies and probed with either anti-AKT or anti-NQO2 antibody. AKT signals were quantified by densitometry analysis and dose-dependent increase of AKT signal was detected in NQO2 immunoprecipitates. (**D**) Analysis of co-localization of NQO2 and AKT in PC-3 cells. Cells were incubated with anti-NQO2 and anti-AKT antibodies, and counter-stained with DAPI for identification of location of cell nucleus. The presence of NQO2 and AKT was identified and visualized by FITC-conjugated or rhodamine-conjugated secondary antibodies, respectively, as described in Materials and Methods. The images were examined by immunofluorescence microscopy. The co-localization of NQO2 (green) with AKT (red) appeared as a yellow color in the merged images.
doi:10.1371/journal.pone.0101070.g001

supports their specific interaction and lends credence to the notion that NQO2 might function as enhancer or suppressor of AKT activity.

Whether NQO2 forms a specific complex with AKT was next examined by gel filtration. CWR22Rv1 cell lysates were subjected to Superdex 200 column chromatography and eluted fractions were analyzed for presence of NQO2 and AKT by immunoblotting. The bulk of NQO2 eluted in fractions 25 to 36, and distinct peaks were observed in fractions 27 to 31. AKT eluted in fractions 24 to 31 (Fig. 1B) which overlapped with NQO2 in fractions 25 to 31. This result shows that a percentage of NQO2 might form a complex with AKT. To additionally test and confirm interaction between NQO2 and AKT, assays involving reciprocal immune-precipitation followed by western blot analysis were performed; constant input of AKT (or NQO2) at 2.5 µg was incubated with 0, 1.0 and 2.5 µg of NQO2 (or AKT). The results showed that NQO2 or AKT was immunoprecipitated by anti-AKT or anti-NQO2, respectively (Fig. 1C) in a dose-dependent manner thereby supporting that NQO2 co-exists and possibly binds and forms a complex with AKT.

Next immunofluorescence microscopy analysis was used to test whether NQO2 co-localizes with AKT, thus offering potential for their interaction in intact cultured cells. PC-3 cells were incubated with anti-AKT and anti-NQO2 antibodies, as described in Materials and Methods. Analysis of the images showed the co-localization of NQO2 (green) with AKT (red) as illustrated by the appearance of yellow colored, merged image (Fig. 1D). These

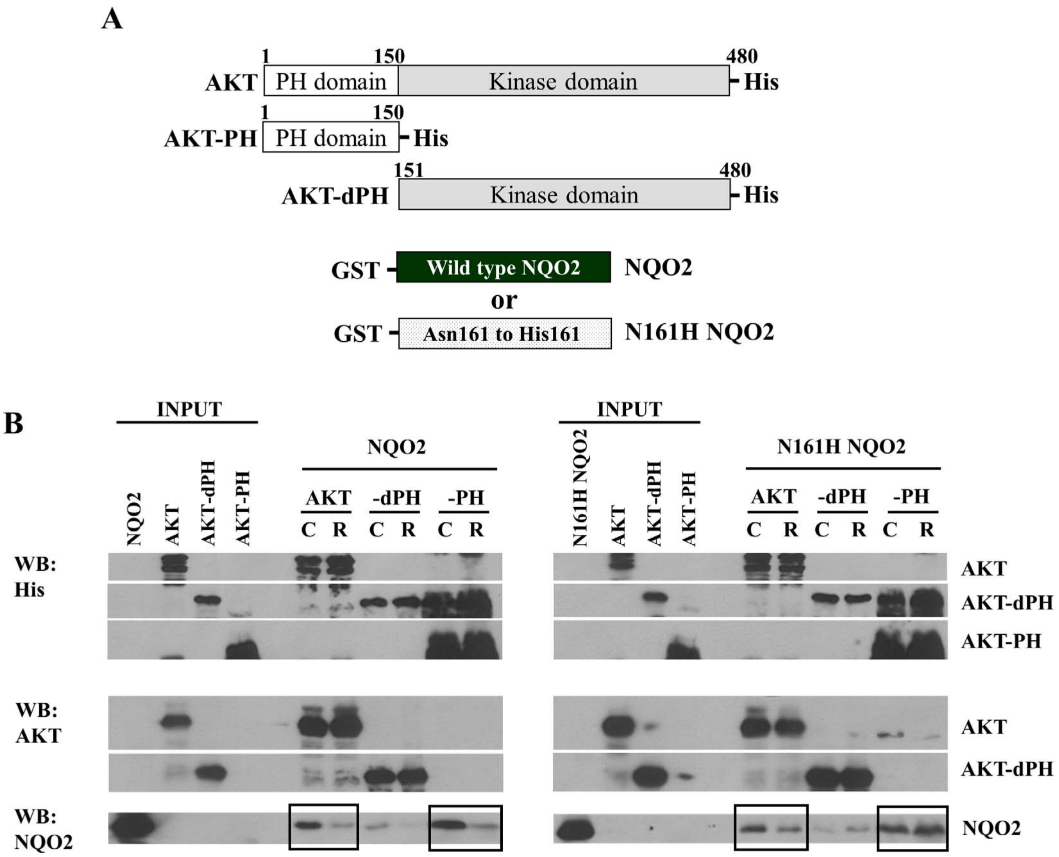

Figure 2. Effects of NQO2 on resveratrol mediated AKT control. (**A**) Mapping of the sites of NQO2 and AKT interaction using full-length and truncated deletion constructs of NQO2 and AKT, respectively. (**B**) Determination of the interactions between NQO2 and AKT by pull-down assays. His-AKT, His-AKT-dPH or His-AKT-PH (up to 2.5 µg) was used to pull down purified GST-NQO2 or GST-N161H NQO2. The bound proteins were subjected to 10% SDS-PAGE and then immunoblotted using monoclonal antibody against AKT, NQO2 or His. Conditions for C (control) and R (resveratrol) consisted of GST-tagged-NQO2 pre-incubated with or without 1 mM resveratrol added prior to performance of the pull down assays.
doi:10.1371/journal.pone.0101070.g002

Table 1. Determination of the binding of AKT activators, Ins(1,3,4,5)P4 (IP4), PtdIns(3,4,5)P3 (PIP3) or phosphatidylserine (PS) to AKT using small molecule docking.

Receptor(s)/Scores	IP4	PIP3 HG (IP4H)	PS
AKT (PDB code [3O96])	−60.65	−100.06	−67.59
AKT with resveratrol	−52.94	−90.01	−65.78
AKT with inhibitor VIII	−33.70	−54.18	−22.60
AKT – NQO2 (PDB code [1SG0])	−61.78	−85.59	−61.15
AKT – NQO2 with resveratrol	−60.20	−87.88	−64.19
AKT with resveratrol – NQO2	−54.91	−85.4	−62.90
AKT with resveratrol – NQO2 with resveratrol	−56.77	−102.22	−76.86
AKT with resveratrol – NQO2 N161H	−55.77	−72.08	−61.37

doi:10.1371/journal.pone.0101070.t001

findings further support that interaction occurs between NQO2 and AKT.

Determination of Site of interaction between NQO2 and AKT and modulation of NQO2:AKT interaction by resveratrol

Protein docking studies predicted that NQO2 interacts with both PH and kinase domains of AKT (Fig. 1A). To independently confirm the predicted AKT binding site of NQO2, full length His-tag AKT or AKT containing either PH or kinase domain were generated and designated as AKT-PH or AKT-dPH (deletion of PH domain), respectively (Fig. 2A). Using pulldown assays and probing reciprocally by immunoblot analysis, it was found that NQO2 interacted with full length or PH domain-containing AKT; in contrast, only a weak interaction was observed between NQO2 and AKT-dPH suggesting that the PH segment of AKT contains structural features that are required for optimal binding and promotion of interaction with NQO2. A less likely explanation is that the kinase domain of AKT interferes with or disrupts the interaction between NQO2 and AKT. Of note, since the PH-containing AKT contained carboxy-terminal His-tag, its status was unable to be probed by AKT antibody (#9272) (Cell Signaling Technology, Inc., Beverly, MA, USA) used in this study; instead, the pulldown results were queried using antibody directed against His-tag. The results showed that different forms of AKT used as inputs were clearly detected by the anti-His-tag antibody used (Fig. 2B). To elucidate the effects of resveratrol on NQO2:AKT interaction, GST-tag NQO2 was generated. These included full length wild type NQO2 and NQO2 with Asn161, the key amino acid for binding resveratrol, mutated to His161 (N161H NQO2) (Fig. 2A). Pre-incubation of resveratrol with wild type NQO2 significantly reduced the extent of binding of NQO2 with full length or PH-containing AKT (Fig. 2B) suggesting that NQO2, once bound with resveratrol, probably assumes a conformational change making it less capable of interaction with AKT. In N161H mutated NQO2, interaction with full length or PH-containing AKT was comparable to wild type NQO2; however, owing to lack of binding of N161H NQO2 to resveratrol, significantly less pronounced effect of resveratrol was observed on the interaction between mutant NQO2 and AKT (Fig. 2B). Taken as a whole, these results suggest that NQO2:AKT can exist intracellularly as a complex and that the avidity of their reciprocal binding may be further modulated by exposure to resveratrol.

How NQO2/AKT interaction affects AKT activation

Since PH domain of AKT is required for binding to NQO2, a question of interest is whether this interaction might disrupt or attenuate AKT activation by interfering with the binding of phosphoinositides to the PH domain of AKT. To test this possibility, small molecular docking analysis was employed using Dock 6. We first focused on AKT binding by its activators, Ins(1,3,4,5)P4 (IP4) and PtdIns(3,4,5)P3 (PIP3). The crystal structure of human AKT (PDB code: 3O96) was used to dock the IP4 and PIP3. Total binding energies were calculated and the predicted binding affinity was compared with AKT in complex with its well established allosteric inhibitor VIII. Results showed that PIP3 binds with higher affinity to AKT compared to IP4; binding of either PIP3 or IP4 was reduced ~45% by the presence of inhibitor VIII (Table 1). Since it has been reported that membrane phosphatidylserine (PS) is also required for AKT activation via interaction with its PH domain [38], we next examined the effects of inhibitor VIII on PS and AKT binding. A ~67% reduction on binding affinity was observed (Table 1). These results support the feasibility and use of *in-silico* approaches to simulate step-by-step intracellular biological events. To investigate whether this approach can be used to ascertain whether interaction of NQO2 and AKT affects AKT activation by disrupting binding of PIP3, IP4, or PS with AKT, AKT-NQO2 complex was first docked by GRAMM-X, using pertinent structures created with Chimera and used as reference receptors (target protein complexes). Total binding energy analysis predicted that when AKT is bound with NQO2, a ~15% reduction occurred in AKT interaction with PIP3; however, no effect on IP4-AKT or PS-AKT interaction was observed (Table 1). These results suggest that NQO2 is most likely a weak intracellular modulator of the PIP3-AKT binding and interaction, albeit possibly an effect that could attenuate the dynamics of AKT activation. Notably, this result also supports our published study showing an increase in AKT activity in NQO2 knockdown shRNA25 cells [19].

Binding and interaction of AKT with resveratrol

Published reports have shown that BCR-ABL kinase inhibitors can bind and inhibit the activity of both non-kinase and kinase targets, NQO2 and ABL, respectively [22,23]. Based on our observation that NQO2 attenuates AKT activity [19], we hypothesize that resveratrol, alone or in complex with NQO2, may serve as a novel modulator of endogenous AKT by fine tuning the control of AKT activation, activity and function. This hypothesis was evaluated using the UCSF's DOCK6.3 approach.

A

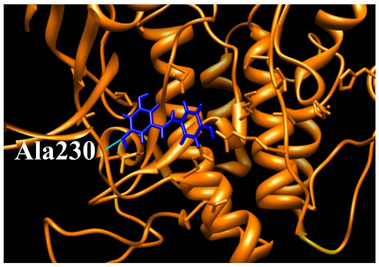

Ligand	GS	VDW	ES
resveratrol	-35.75	-33.96	-1.79
ATP	-48.47	-45.96	-2.51

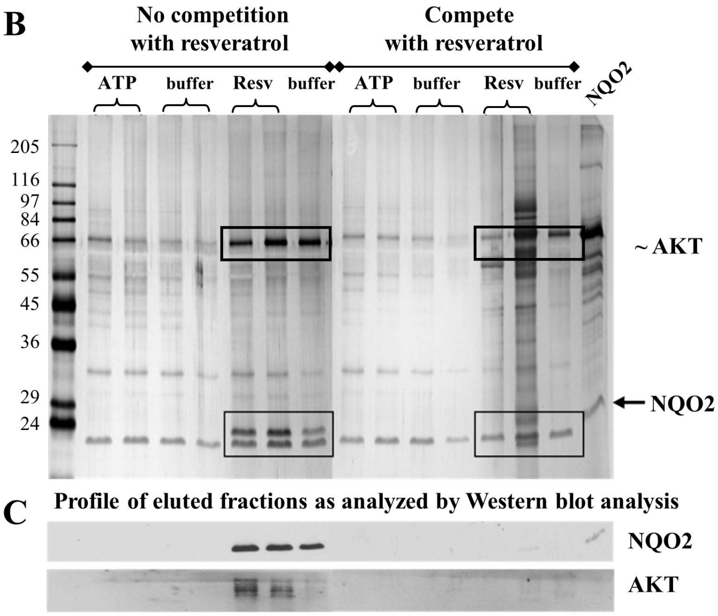

D

Receptor(s)/Ligand	PPI Score
AKT – NQO2	-31.63
AKT – NQO2 with resveratrol	-19.22
AKT with resveratrol – NQO2	-26.24
AKT with resveratrol – NQO2 with resveratrol	-6.16
AKT with resveratrol – NQO2 N161H	-24.14

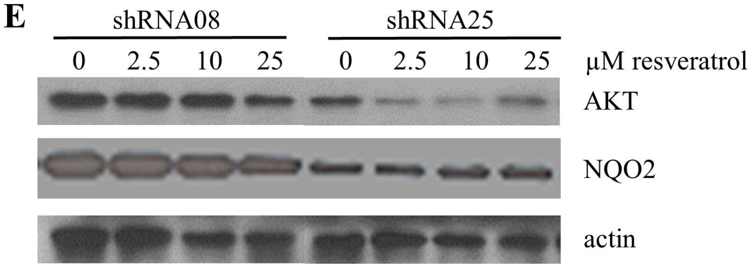

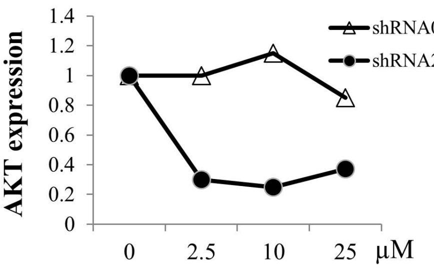

Figure 3. Binding of resveratrol to AKT. (**A**) Docking of resveratrol to AKT (top panel) using DOCK 6.3 program. The grid score (GS) represented the sum of the van der Waals (VDW) and electrostatic scores (ES); compounds showing low GS are likely to have a high affinity towards the receptor (targeting protein). The affinity of resveratrol and ATP to AKT was compared and shown in lower panel. (**B**) Binding of specific proteins from K562 cell extracts to immobilized resveratrol affinity columns and isolation of resveratrol target proteins. Cell lysates fractionated on control and resveratrol

affinity columns were eluted with NaCl, ATP and resveratrol. Silver stained protein profiles of the ATP and resveratrol eluted fractions without (left side of the gel) and with (right side of the gel) competition by resveratrol were shown. (**C**) Western blot analysis examined the elution profiles and demonstrated the presence of AKT and NQO2 in resveratrol eluted fractions in column separation performed without prior competition of cell lysates with resveratrol. (**D**) Effect of resveratrol on interaction and complex formation between NQO2 and AKT was evaluated by PatchDock and scored by FireDock. (**E**) Changes on AKT protein expression by resveratrol were examined in control shRNA08 and NQO2 knockdown shRNA25 cells. Cells were treated with increasing doses of resveratrol (0, 2.5, 10 and 25 μM) for 72 h and changes on AKT were analyzed by western blot analysis. The blots were also probed for actin which served as loading control.
doi:10.1371/journal.pone.0101070.g003

ATP binding site of AKT was used as a receptor for docking resveratrol and the results were visualized using Chimera accessory programs. Evidently, resveratrol fits well within the ATP pocket of AKT (Fig. 3A) suggesting that AKT is a potential resveratrol target protein. This possibility was strengthened by the predicted binding of resveratrol to the active site of AKT, based on the calculated grid score (GS, indicative of overall binding energy), van der Waals energy (VDW) and electrostatic energy (ES) (Fig. 3A).

That AKT is a resveratrol target was further examined by resveratrol-appended affinity column chromatography using K562 cell extracts. The results showed that most proteins were loosely retained on the affinity column and only a few, distinct silver stained protein bands were eluted with resveratrol (Fig. 3B). Immunoblot analysis on fractions eluted using buffers containing increasing salt concentration, ATP, or resveratrol showed that the column-bound NQO2 or AKT did not elute with buffer supplemented with ATP, and was only displaced from the affinity column by resveratrol (Fig. 3C). Authenticity and binding affinity of AKT as a prospective resveratrol target protein to the affinity column was confirmed by competition with excess resveratrol prior to affinity column fractionation, which effectively competed binding of both NQO2 and AKT (Fig. 3C). These results indicate that AKT binds tightly to resveratrol, in line with its classification as a novel resveratrol target protein. The inability of column-bound AKT to be displaced by ATP (Fig. 3C) was unexpected since, in computer modeling analysis, ATP had lower binding energy (hence higher affinity), more favorable VDW's forces compared to resveratrol (Fig. 3A). One possible explanation for this observation is binding of AKT to resveratrol may be accompanied by a conformational change rendering AKT less accessible to ATP. Additionally, this effect may well be applicable to the control of AKT activity by resveratrol. Studies are planned to further investigate these regulatory aspects of AKT by resveratrol.

Since resveratrol can independently bind to either NQO2 or AKT, the effect of resveratrol on AKT/NQO2 interaction was additionally studied by protein-protein docking. The docking score obtained showed essentially no effect of pre-binding of AKT by resveratrol on its subsequent interaction with NQO2 (Fig. 3D). In striking contrast, pre-binding of NQO2 resulted in a significant reduction in its interaction with AKT (Fig. 3D). Not surprisingly, pre-binding with resveratrol using both NQO2 and AKT also markedly impaired their subsequent interaction (Fig. 3D) further supporting that binding of resveratrol to NQO2 is likely to be the fast and rate determining step in governing AKT:NQO2 interaction. To further understand how NQO2 contributes to resveratrol-induced AKT control, NQO2 expressing and knock-down cells were treated with different dose of resveratrol, followed by western blot analysis. These studies showed that while 2.5 μM resveratrol sufficed to down regulate AKT protein expression in NQO2 knockdown shRNA25 cells, a 10-fold increase in resveratrol (25 μM) failed to cause a comparable decrease in AKT levels in control shRNA08 cells (Fig. 3E). These results suggest another level of control by resveratrol and NQO2 interplay impinging on control of AKT expression further supporting the role of resveratrol in co-targeting the dynamic regulation of AKT and NQO2.

The change on NQO2 levels following resveratrol treatments was also examined. As expected, a ~50% decrease in NQO2 expression was observed in knockdown shRNA25 cells relative to shRNA08 cells; however, exposure to resveratrol had no effect on NQO2 in either cells (Fig. 3E), suggesting that control of NQO2 is distinct from regulation of AKT by resveratrol.

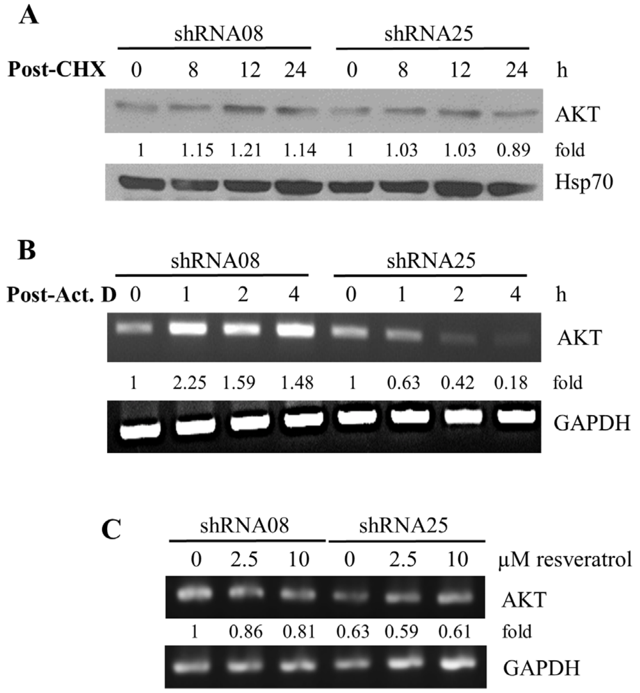

Figure 4. Analysis of stability of AKT protein and mRNA in relation to knockdown status of NQO2. (**A**) Turnover/degradation of AKT in shRNA08 or shRNA25 cells. Cells were treated with 30 μg/ml CHX to inhibit new protein synthesis for the times indicated. Changes in AKT was analyzed by western blot analysis, with Hsp70 expression used as loading control. AKT expression was quantified by densitometry analysis and the expression was presented as fold relative to time zero. (**B**) Effect of knockdown NQO2 on AKT mRNA stability. Both shRNA08 and shRNA25 cells were treated with transcription inhibitor actinomycin D (5 μg/ml). Cells were harvested at 0, 1, 2 and 4 h after treatment. Total cellular RNA was isolated and residual mRNAs were determined by RT-PCR, using GAPDH mRNA expression as internal control. AKT expression was quantified by densitometric analysis and the expression was presented as fold relative to time zero. (**C**) Effect of resveratrol on AKT mRNA. Cells (shRNA08 and shRNA25 cells) were treated with increasing doses of resveratrol (0, 2.5 and 10 μM) for 72 h and changes on AKT mRNA levels were assayed by RT-PCR, quantified by densitometry, and expressed as fold differences by normalization against GAPDH.
doi:10.1371/journal.pone.0101070.g004

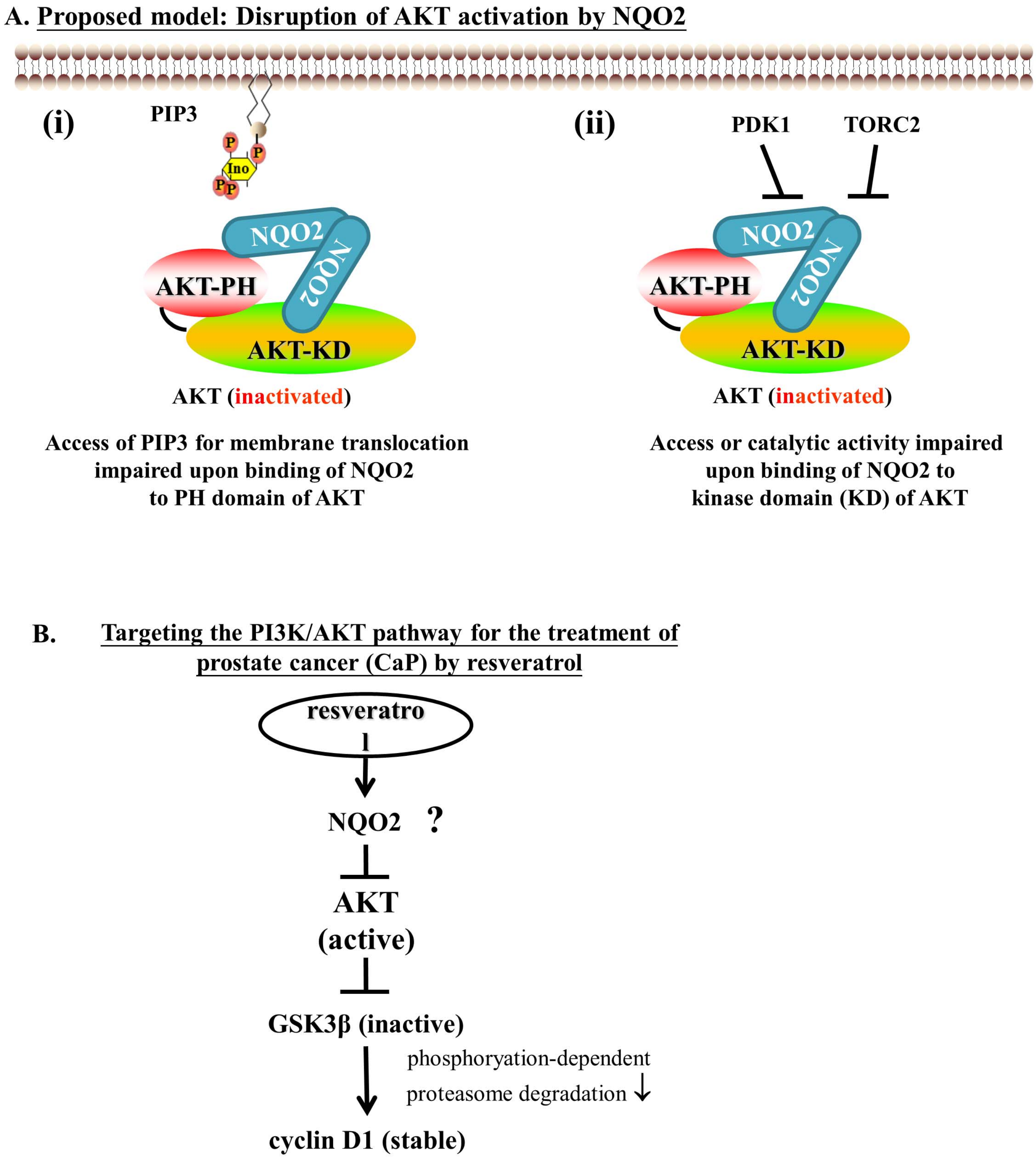

Figure 5. A proposed mechanism on the role of NQO2 in regulating AKT and AKT-mediated signaling events. (**A**) Hypotheses: NQO2 acts as a physiologic partner and modulator of AKT by disruption of its activation. NQO2/AKT interaction may (i) disrupt or limit the access of PIP3 for membrane translocation by AKT or (ii) inhibit the catalytic activity of AKT. (**B**) NQO2 regulates GSK-3β-mediated cyclin D1 degradation by binding AKT and/or inhibiting its phosphorylation-dependent activation. This sequence of events is effectively attenuated in NQO2 knockdown cells or when cells are exposed to resveratrol, which acts by effectively binding and sequestrating NQO2, rendering it incapable of forming a complex with AKT.
doi:10.1371/journal.pone.0101070.g005

Effect of NQO2 knockdown on AKT expression

To obtain information on how NQO2 might contribute to the differential expression of AKT observed in resveratrol-treated control shRNA08 and NQO2 knockdown shRNA25 cells (Fig. 3E), the stability of AKT was determined. In a time course experiment, control and NQO2 knockdown cells were first incubated with a

protein synthesis inhibitor, cycloheximide (CHX), and cells were harvested at different times post treatment. Extracts were prepared and Western blot analysis was performed to assess AKT expression using Hsp70 level as a loading control. Results in Fig. 4A showed a similar, stable AKT protein half-life in both cells, suggesting that NQO2 do not directly participate in control of turnover of AKT protein. We next tested whether NQO2 status affected transcriptional control of AKT. Control shRNA08 and NQO2 knockdown shRNA25 cells were exposed to actinomycin D (Act. D); cells were harvested at different times and changes in AKT mRNA levels were assayed by RT-PCR. As shown in Figure 4B, the half-life of AKT mRNA decreased from ~4 h in control cells to ~1 h in NQO2 knockdown cells, showing that AKT mRNA was several fold more stable in control cells (shRNA08) compared to NQO2 knockdown cells (shRNA25). We also determined whether resveratrol treatment altered AKT mRNA expression. NQO2 knockdown is accompanied by ~47% reduction in AKT mRNA level. Exposure to 2.5 and 10 μM resveratrol reduced AKT mRNA by 14% and 19% in control shRNA08 cells but had no effect in NQO2 knockdown shRNA25 cells (Fig. 4C). These results suggest that NQO2 and resveratrol exert a complex effect in the control of AKT, viz., a NQO2-dependent transcriptional control, as supported by the substantial decrease in half-life of AKT mRNA in cells harboring partial silencing of NQO2, and a post-transcriptional effect attributed to treatment by resveratrol, by an NQO2-independent mechanism.

Discussion

Experimental studies corroborated by clinical evidence point to deregulated AKT kinase activity as being causally linked to oncogenic transformation and tumor growth, thus providing the impetus for understanding the regulation of AKT as a critical target for the management and therapy of malignant diseases. Resveratrol has been reported to inhibit AKT activity; however, little is known about the underlying mechanism. We previously identified NQO2 as a resveratrol target protein [18] and showed that NQO2 is involved in resveratrol mediated AKT/GSK-3β/cyclin D1 control [19]. How NQO2 controls AKT remains largely unknown. Thus our objectives in this study are to use multidisciplinary approaches to gain information on role resveratrol and NQO2 play in the control of AKT.

A lack of knowledge of three dimensional (3D) structure of NQO2:AKT complex limited efforts for detailed analysis of binding activity and specificity. Accordingly we performed *in silico* computer modeling studies to begin the exploration of the structure-activity relationship between NQO2 and AKT with its ligands (modulating small molecules). The *in-silico* simulations were aimed at uncovering possible interactive scenarios existing and occurring in the cell that might be amenable to correlative experimental analysis. To these ends, 3D structures of NQO2:AKT complex were first generated by GRAMM-X molecular modeling and used to probe the nature of docking between these two proteins, with and without the participation of ligands such as resveratrol. It was reasoned that this approach might also be applicable for the analysis of interaction between AKT and AKT:NQO2 complex with known AKT activators or inhibitors, e.g., PIPs, PS, and inhibitor VIII. To analyze the likelihood of interaction between AKT:NQO2, beyond qualitative physical measurements revealed by GRAMM-X protein docking, PatchDock/FireDock was employed to generate scores that show quantitatively protein:protein binding as well as the ability to dock with ligands [35–37]. Interestingly, protein-protein docking scores revealed that pre-binding of NQO2 with resveratrol reduced by ~40% affinity between AKT:NQO2 (Fig. 3D) whereas pre-binding of both AKT and NQO2 with resveratrol resulted in ~80% decrease in its binding affinity (Fig. 3D). In contrast, pre-binding of resveratrol to either NQO2 or AKT did not affect the binding affinity of PIP3 to AKT:NQO2 complex compared to AKT alone (Table 1). These results suggest that resveratrol will not likely augment NQO2-mediated AKT deactivation in NQO2-expressing cells. Conceivably, however, resveratrol could deactivate AKT at high concentration via direct binding and therefore offer the potential for adjunctive therapy in individuals known to harbor AKT inhibitor resistant cells. Taken together, the computer modeling studies in this study clearly predicted binding and interaction existing between NQO2 and AKT and that this interaction is subject to modulation by ligands such as resveratrol and by AKT activators or inhibitors. In principle, therefore, this approach may be expanded into searches for more potent and specific modulators and or activators/inhibitors for the control AKT activity or interaction between the AKT:NQO2 complex.

AKT has been shown to promote cancer cell proliferation and survival by diverse mechanisms including the activation of downstream targets including forkhead transcription factors, GSK3, BAD, Bcl-XL, nuclear factor-kB, and mammalian target of rapamycin (mTOR) kinase [5,6,39,40]. In our previous studies we found that NQO2 is involved in GSK-3β-mediated cyclin D1 degradation as well as in control of AKT activity [19]. In the current study, we have expanded these studies using leads revealed by computer modeling analysis. We have found that NQO2 suppression of AKT may be explained in part by NQO2 targeting both the PH and KD domains of AKT and that the NQO2:AKT interaction interferes with binding between PH domain of AKT with its activator PIP3 (Table 1), posibly impinging on AKT activation and activity. This observation may have broader implications based on the flurry of activities for developing AKT inhibitors targeted specifically against the PH domain of AKT [41]; thus, NQO2 may harness the potential as a novel endogenous cellular protein that may well modulate as well complement chemotherapeutic agents designed as AKT PH domain-specific inhibitors.

The development of selective, potent inhibitors targeting the ATP-binding pocket of AKT [42] has been hampered by the extensive homology in ATP-binding sites among kinases. In this study we found that resveratrol binds AKT at its ATP site with lower binding affinity compared to ATP (Fig. 3A); curiously, AKT bound to resveratrol failed to be displaced by ATP (Fig. 3C) in resveratrol affinity columns. This observation raises the tantalizing possibility that resveratrol might be proffered as a low efficient, ATP-competitive AKT inhibitor with a reduced likelihood for inducing therapeutic resistance. Another equally attractive alternative is that resveratrol might function as a heterotropic allosteric modulator of AKT, vis-à-vis, by binding to AKT at a site distinct from the active site and effecting a decrease in binding affinity at the active site between ATP and AKT, rendering the enzyme inactive. Studies to test these possibilities in the context of resveratrol serving as allosteric modulator of AKT are underway in our laboratory.

It is noteworthy that the novel NQO2-non-kinase-mediated AKT control unraveled in this study differs significantly from traditional PTEN/PI3K mediated AKT control [43–45]; namely, the inhibition of AKT by over-expressing NQO2 in PTEN-deficient cancer cells can be expected to inhibit tumor growth. This yet-to-be-explored possibility might consolidate the notion that NQO2 indeed functions as a tumor suppressor in PTEN-deficient cancer cells. Alternatively, given that both NQO2 and PTEN can negatively regulate PI3K/AKT pathway via control of

the interaction between AKT and its activator PIP3, an increase in NQO2 expression could restrict proliferation and survival of PTEN-deficient cancer cells by amplifying sensitivity to chemotherapeutic agents. A hypothetic model showing how NQO2 works in control of AKT activation is illustrated in Figure 5. Mechanistic possibilities depicted include: (i) contribution of resveratrol in control of PI3K/AKT signaling in cancer cells *in vitro* and possibly *in vivo*; (ii) role of NQO2 in the control of AKT activation (Fig. 5A) and additional modulation of this control by resveratrol (Fig. 5B); and (iii) whether NQO2 might function as a tumor suppressor in PTEN-deficient cancer cells by the novel NQO2-non-kinase-mediated PI3K/AKT survival pathway.

In summary, the control of AKT by NQO2 revealed by the present studies extends our previous report on the control of cyclin D1 by NQO2 mediated control of GSK-3β [19] and provides new insights on our understanding of how NQO2 controls cyclin D1 via AKT/GSK-3β signaling. Thus, binding and complex formation between NQO2 and AKT in the cytosol could restrict and prevent binding of AKT with its activator PIP3, effectively suppressing phosphorylation-dependent activation of AKT which in turn interferes with phosphorylation of GSK-3β, subsequently suppressing GSK-3β-mediated cyclin D1 degradation by proteasomes. In a larger context, results of this study validate the chemotherapeutic potential of targeting AKT by NQO2, and that NQO2 acts as a versatile AKT inhibitor affecting its ATP-binding pocket, the PH domain, and disrupts its interaction with upstream inhibitors interfering with enzyme activation of PTEN. From a public health perspective, control of AKT by NQO2 has implications for treating PTEN-deficient cells and circumventing drug resistance to AKT in cells harboring over-expression of NQO2.

Author Contributions

Conceived and designed the experiments: TCH. Performed the experiments: TCH CYL. Analyzed the data: TCH DJB CYL. Wrote the paper: TCH EW JMW.

References

1. Chan TO, Tsichlis PN (2001) PDK2: a complex tail in one Akt. Sci STKE 2001: pe1.
2. Vanhaesebroeck B, Alessi DR (2000) The PI3K-PDK1 connection: more than just a road to PKB. Biochem J 346 Pt 3: 561–576.
3. Galetic I, Andjelkovic M, Meier R, Brodbeck D, Park J, et al. (1999) Mechanism of protein kinase B activation by insulin/insulin-like growth factor-1 revealed by specific inhibitors of phosphoinositide 3-kinase–significance for diabetes and cancer. Pharmacol Ther 82: 409–425.
4. Brazil DP, Hemmings BA (2001) Ten years of protein kinase B signalling: a hard Akt to follow. Trends Biochem Sci 26: 657–664.
5. Cantley LC, Neel BG (1999) New insights into tumor suppression: PTEN suppresses tumor formation by restraining the phosphoinositide 3-kinase/AKT pathway. Proc Natl Acad Sci U S A 96: 4240–4245.
6. Vivanco I, Sawyers CL (2002) The phosphatidylinositol 3-Kinase AKT pathway in human cancer. Nat Rev Cancer 2: 489–501.
7. Di Cristofano A, Pandolfi PP (2000) The multiple roles of PTEN in tumor suppression. Cell 100: 387–390.
8. Blando J, Portis M, Benavides F, Alexander A, Mills G, et al. (2009) PTEN deficiency is fully penetrant for prostate adenocarcinoma in C57BL/6 mice via mTOR-dependent growth. Am J Pathol 174: 1869–1879.
9. Li R, Dai H, Wheeler TM, Sayeeduddin M, Scardino PT, et al. (2009) Prognostic value of Akt-1 in human prostate cancer: a computerized quantitative assessment with quantum dot technology. Clin Cancer Res 15: 3568–3573.
10. de Souza PL, Russell PJ, Kearsley J (2009) Role of the Akt pathway in prostate cancer. Curr Cancer Drug Targets 9: 163–175.
11. Taylor BS, Schultz N, Hieronymus H, Gopalan A, Xiao Y, et al. (2010) Integrative genomic profiling of human prostate cancer. Cancer Cell 18: 11–22.
12. Ghosh PM (2012) What controls PTEN and what it controls (in prostate cancer). Asian J Androl 14: 130–131.
13. Benitez DA, Pozo-Guisado E, Clementi M, Castellon E, Fernandez-Salguero PM (2007) Non-genomic action of resveratrol on androgen and oestrogen receptors in prostate cancer: modulation of the phosphoinositide 3-kinase pathway. Br J Cancer 96: 1595–1604.
14. Sexton E, Van Themsche C, LeBlanc K, Parent S, Lemoine P, et al. (2006) Resveratrol interferes with AKT activity and triggers apoptosis in human uterine cancer cells. Mol Cancer 5: 45.
15. Aziz MH, Nihal M, Fu VX, Jarrard DF, Ahmad N (2006) Resveratrol-caused apoptosis of human prostate carcinoma LNCaP cells is mediated via modulation of phosphatidylinositol 3′-kinase/Akt pathway and Bcl-2 family proteins. Mol Cancer Ther 5: 1335–1341.
16. Hudson TS, Hartle DK, Hursting SD, Nunez NP, Wang TT, et al. (2007) Inhibition of prostate cancer growth by muscadine grape skin extract and resveratrol through distinct mechanisms. Cancer Res 67: 8396–8405.
17. Gill C, Walsh SE, Morrissey C, Fitzpatrick JM, Watson RW (2007) Resveratrol sensitizes androgen independent prostate cancer cells to death-receptor mediated apoptosis through multiple mechanisms. Prostate 67: 1641–1653.
18. Buryanovskyy L, Fu Y, Boyd M, Ma Y, Hsieh TC, et al. (2004) Crystal structure of quinone reductase 2 in complex with resveratrol. Biochemistry 43: 11417–11426.
19. Hsieh TC, Yang CJ, Lin CY, Lee YS, Wu JM (2012) Control of stability of cyclin D1 by quinone reductase 2 in CWR22Rv1 prostate cancer cells. Carcinogenesis 33: 670–677.
20. Diehl JA, Cheng M, Roussel MF, Sherr CJ (1998) Glycogen synthase kinase-3beta regulates cyclin D1 proteolysis and subcellular localization. Genes Dev 12: 3499–3511.
21. Alt JR, Cleveland JL, Hannink M, Diehl JA (2000) Phosphorylation-dependent regulation of cyclin D1 nuclear export and cyclin D1-dependent cellular transformation. Genes Dev 14: 3102–3114.
22. Rix U, Hantschel O, Durnberger G, Remsing Rix LL, Planyavsky M, et al. (2007) Chemical proteomic profiles of the BCR-ABL inhibitors imatinib, nilotinib, and dasatinib reveal novel kinase and nonkinase targets. Blood 110: 4055–4063.
23. Winger JA, Hantschel O, Superti-Furga G, Kuriyan J (2009) The structure of the leukemia drug imatinib bound to human quinone reductase 2 (NQO2). BMC Struct Biol 9: 7.
24. Hsieh TC (2009) Uptake of resveratrol and role of resveratrol-targeting protein, quinone reductase 2, in normally cultured human prostate cells. Asian J Androl 11: 653–661.
25. Hsieh TC (2009) Antiproliferative effects of resveratrol and the mediating role of resveratrol targeting protein NQO2 in androgen receptor-positive, hormone-non-responsive CWR22Rv1 cells. Anticancer Res 29: 3011–3017.
26. Wang Z, Hsieh TC, Zhang Z, Ma Y, Wu JM (2004) Identification and purification of resveratrol targeting proteins using immobilized resveratrol affinity chromatography. Biochem Biophys Res Commun 323: 743–749.
27. Hsieh TC, Wang Z, Deng H, Wu JM (2008) Identification of glutathione sulfotransferase-pi (GSTP1) as a new resveratrol targeting protein (RTP) and studies of resveratrol-responsive protein changes by resveratrol affinity chromatography. Anticancer Res 28: 29–36.
28. Wessel D, Flugge UI (1984) A method for the quantitative recovery of protein in dilute solution in the presence of detergents and lipids. Anal Biochem 138: 141–143.
29. Wang Z, Chen Y, Labinskyy N, Hsieh TC, Ungvari Z, et al. (2006) Regulation of proliferation and gene expression in cultured human aortic smooth muscle cells by resveratrol and standardized grape extracts. Biochem Biophys Res Commun 346: 367–376.
30. Pettersen EF, Goddard TD, Huang CC, Couch GS, Greenblatt DM, et al. (2004) UCSF Chimera–a visualization system for exploratory research and analysis. J Comput Chem 25: 1605–1612.
31. Tovchigrechko A, Vakser IA (2006) GRAMM-X public web server for protein-protein docking. Nucleic Acids Res 34: W310–314.
32. Tovchigrechko A, Vakser IA (2005) Development and testing of an automated approach to protein docking. Proteins 60: 296–301.
33. Sanner MF, Olson AJ, Spehner JC (1996) Reduced surface: an efficient way to compute molecular surfaces. Biopolymers 38: 305–320.
34. Hsieh TC, Wong C, John Bennett D, Wu JM (2011) Regulation of p53 and cell proliferation by resveratrol and its derivatives in breast cancer cells: an in silico and biochemical approach targeting integrin alphavbeta3. Int J Cancer 129: 2732–2743.
35. Schneidman-Duhovny D, Inbar Y, Nussinov R, Wolfson HJ (2005) PatchDock and SymmDock: servers for rigid and symmetric docking. Nucleic Acids Res 33: W363–367.
36. Andrusier N, Nussinov R, Wolfson HJ (2007) FireDock: fast interaction refinement in molecular docking. Proteins 69: 139–159.
37. Mashiach E, Schneidman-Duhovny D, Andrusier N, Nussinov R, Wolfson HJ (2008) FireDock: a web server for fast interaction refinement in molecular docking. Nucleic Acids Res 36: W229–232.
38. Huang BX, Akbar M, Kevala K, Kim HY (2011) Phosphatidylserine is a critical modulator for Akt activation. J Cell Biol 192: 979–992.
39. Altomare DA, Testa JR (2005) Perturbations of the AKT signaling pathway in human cancer. Oncogene 24: 7455–7464.

40. Hennessy BT, Smith DL, Ram PT, Lu Y, Mills GB (2005) Exploiting the PI3K/AKT pathway for cancer drug discovery. Nat Rev Drug Discov 4: 988–1004.
41. Barnett SF, Defeo-Jones D, Fu S, Hancock PJ, Haskell KM, et al. (2005) Identification and characterization of pleckstrin-homology-domain-dependent and isoenzyme-specific Akt inhibitors. Biochem J 385: 399–408.
42. Luo Y, Shoemaker AR, Liu X, Woods KW, Thomas SA, et al. (2005) Potent and selective inhibitors of Akt kinases slow the progress of tumors in vivo. Mol Cancer Ther 4: 977–986.
43. Davies MA, Koul D, Dhesi H, Berman R, McDonnell TJ, et al. (1999) Regulation of Akt/PKB activity, cellular growth, and apoptosis in prostate carcinoma cells by MMAC/PTEN. Cancer Res 59: 2551–2556.
44. Jetzt A, Howe JA, Horn MT, Maxwell E, Yin Z, et al. (2003) Adenoviral-mediated expression of a kinase-dead mutant of Akt induces apoptosis selectively in tumor cells and suppresses tumor growth in mice. Cancer Res 63: 6697–6706.
45. Davies MA, Lu Y, Sano T, Fang X, Tang P, et al. (1998) Adenoviral transgene expression of MMAC/PTEN in human glioma cells inhibits Akt activation and induces anoikis. Cancer Res 58: 5285–5290.
